# Successful therapy of *Candida pulcherrima* fungemia in a premature newborn with liposomal amphotericin B and micafungin

**DOI:** 10.1016/j.mmcr.2016.08.002

**Published:** 2016-08-03

**Authors:** Alexandra Mpakosi, Maria Siopi, Vasiliki Falaina, Nikolaos Siafakas, Emmanuel Roilides, Maria Kimouli, Martha Theodoraki, Paraskevi Karle, Joseph Meletiadis

**Affiliations:** a Department of Microbiology, General Hospital of Nikaia Agios Panteleimon, Athens 18454, Greece; b Clinical Microbiology Laboratory, Attikon University Hospital, Medical School, National and Kapodistrian University of Athens, Athens 12642, Greece; c Neonatal Intensive Care Unit, General Hospital of Nikaia Agios Panteleimon, Athens 18454, Greece; d Infectious Diseases Unit, 3rd Department of Pediatrics, Hippokration Hospital, Thessaloniki 54642, Greece

**Keywords:** Candidemia, Neonate, Combination antifungal therapy, Micafungin, Liposomal amphotericin B

## Abstract

New *Candida* species may cause bloodstream infections challenging current therapeutic approaches because of unpredictable susceptibility and virulence. In the present report, we describe a fungemia case due to *Candida pulcherrima* in a premature neonate. After full *in vitro* diagnostic workup, the neonate was successfully treated with liposomal amphotericin B and micafungin achieving rapid fungal eradication from blood.

## 1 Introduction

*Candida* species are the third most frequently isolated pathogens from blood cultures in neonatal late-onset sepsis (9–13%) [1]. The mortality rate due to *Candida* sepsis is high ranging from 25% to 54% and it can reach 70% in very low birth weight newborns [1], [2]. *Candida albicans* has been historically the most frequent pathogen in neonates followed by *Candida parapsilosis* and other *Candida* species such as *Candida tropicalis*, *Candida glabrata* and *Candida krusei* [3], [4]. However, rare *Candida* species have been increasingly recognized as potential pathogens for neonates [5]. Admission into a Neonatal Intensive Care Unit quadruples the risk of infection by these pathogens [6]. Given the recognition of increased number of bloodstream infections by uncommon opportunistic yeasts with variable susceptibility to antifungal drugs [7], identification of new potential pathogens is important for initiation of prompt and targeted antifungal therapy.

The most recent guidelines by ESCMID favor the use of amphotericin B (conventional and liposomal), fluconazole and micafungin (B-II) for the treatment of neonatal candidemia [8]. However, antifungal resistance to fluconazole is increased among *Candida* non-*albicans* species and particularly *C. glabrata*, whereas echinocandin resistance among *C. glabrata* isolates poses a therapeutic challenge in the treatment of candidemia [9]. While *C. albicans* and *C. parapsilosis* constitute the great majority of *Candida* species causing neonatal candidiasis, rare yeasts with variable susceptibility can occasionally be found and require special care [10].

In the present case report, we describe a rare case of fungemia by *Candida pulcherrima* in a premature neonate together with the diagnostic and therapeutic approaches followed.

## 2 Case

A male newborn born as a gemini B twin with a gestation age of 33 weeks was admitted to the Neonatal Intensive Care Unit at the General Hospital of Nikaia, Athens, Greece due to prematurity and respiratory distress syndrome. The neonate was delivered via spontaneous vaginal delivery following premature rupture of the amniotic membrane. The birth weight was 2080 g. He was initially treated empirically with ampicillin and gentamicin. All drugs were administered via a peripheral catheter, which was changed every three days. Parenteral nutrition was administered until day 3.

On day 0 he developed symptoms and sign of sepsis with fever to 38°C, paleness, indolence and acrocyanosis. His laboratory results demonstrated thrombocytopenia (min 21,000/mm^3^) and increased CRP (max 51 mg/L). The antibiotic therapy was modified to meropenem and teicoplanin and on day 3 liposomal amphotericin B (7 mg/kg/d. i.v.) was added and maintained throughout the treatment after fungal growth was detected in four aerobic blood bottles (BacT Alert, Biomerieux, France) collected on day 0. *C. pulcherrima* was identified as described below and it was detected in all blood cultures collected on days 3, 6 and 7. Ultrasound of the head and abdomen, lumbar puncture, urine culture, ophthalmologic exam and echocardiogram did not indicate disseminated candidiasis.

Four days after initiation of liposomal amphotericin B, the blood cultures remained positive for the same yeast and micafungin 10 mg/kg/d i.v. was added on day 7. His general condition was improved progressively, CRP levels decreased (<3 mg/L) and after two days of combined antifungal therapy on day 9 the blood cultures became negative. The treatment continued for another 16 days. On day 30, the neonate was discharged from the hospital in good condition and with normal laboratory results.

### 2.1 Species identification

**Fig. 1:**
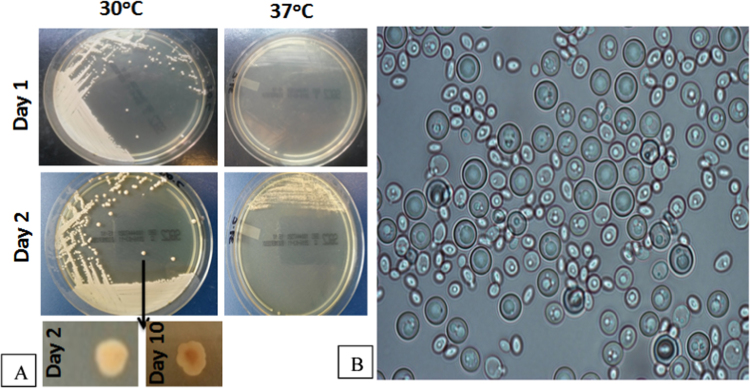
Macroscopic (A) and microscopic (B) photos of *C. pulcherrima*. A. Cream colored colonies with reddish pigment on the reverse of Sabouraud Dextrose Agar. B. Chlamydospores, budding yeast and no pseudohyphae were observed in corn-meal agar after incubation at 37 °C for 48 h. (Magnification 400×).

**Fig. 2:**
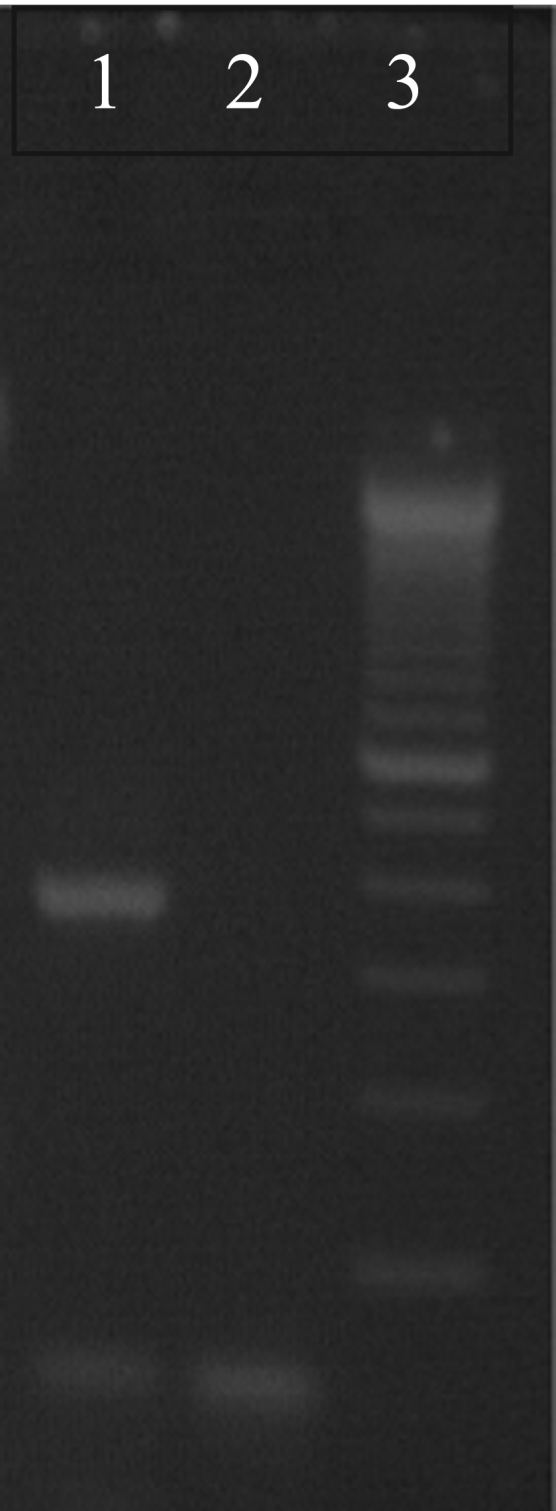
Gel electrophoresis of PCR products after amplification of *C. pulcherrima* DNA with ITS1 (5-TCCGTAGGTGAACCTGCGG-3) and ITS4 (5-TCCTCCGCTTATTGATATGC-3) primers, 1: *C. pulcherrima* 368 bp PCR product, 2: negative control, 3: 100 bp DNA ladder.

The isolate grew on Sabouraud Dextrose agar plates slowly at 37 °C and best at 25–30 °C (Fig. 1A). The colonies were slow growing, convex, cream colored with a reddish pigment developed after 48 h (Fig. 1A). Microscopically ovoid to ellipsoidal budding yeasts with chlamydospores but no pheudohyphae were found (Fig. 1B). Biochemical identification with VITEK 2 Compact automated system (Biomerieux, France) revealed *C. pulcherrima* (good identification with 90% confidence level). Identification was confirmed with ITS sequencing as previously described using ITS1 (5- TCCGTAGGTGAACCTGCGG-3), and ITS4 (5-TCCTCCGCTTATTGATATGC-3) primers (Fig. 2) [11]. High sequence alignment (99%) was found in Genbank Blast analysis with *Metschnikowia pulcherrima*, the sexual name of *C. pulcherrima* (GenBank Accession No KX276090).

### 2.2 *In vitro* susceptibility testing

*In vitro* antifungal susceptibility was tested with Sensititre YeastOne and the minimal inhibitory concentrations (MICs) were for amphotericin B 0.5 mg/L, for fluconazole 0.25 mg/L, for itraconazole 0.03 mg/L, for voriconazole ≤0.008 mg/L, for posaconazole ≤0.008 mg/L, for flucytosine ≤0.06 mg/L, for micafungin 0.12 mg/L, for anidulafungin 0.25 mg/L and for caspofungin 0.5 mg/L. *In vitro* susceptibility to amphotericin B and micafungin were verified with the EUCAST method [12] with 24/48 h MIC of <=0.03/0.125 mg/L and 0.015/0.06 mg/L at 37 °C, and 0.06/0.125 mg/L and 0.06/0.25 mg/L at 30 °C, respectively. The MIC of liposomal amphotericin B was one two-fold dilution lower. The minimal fungicidal concentration was determined as the lowest concentration with no viable cells after subculturing 100 μL from the clear (no visible growth) wells after 48 h at 30 °C and they were 0.5 mg/L for amphotericin B and >8 mg/L for micafungin. No killing was observed at concentrations ≤0.125 mg/L.

### 2.3 *In vitro* combination testing

**Fig. 3:**
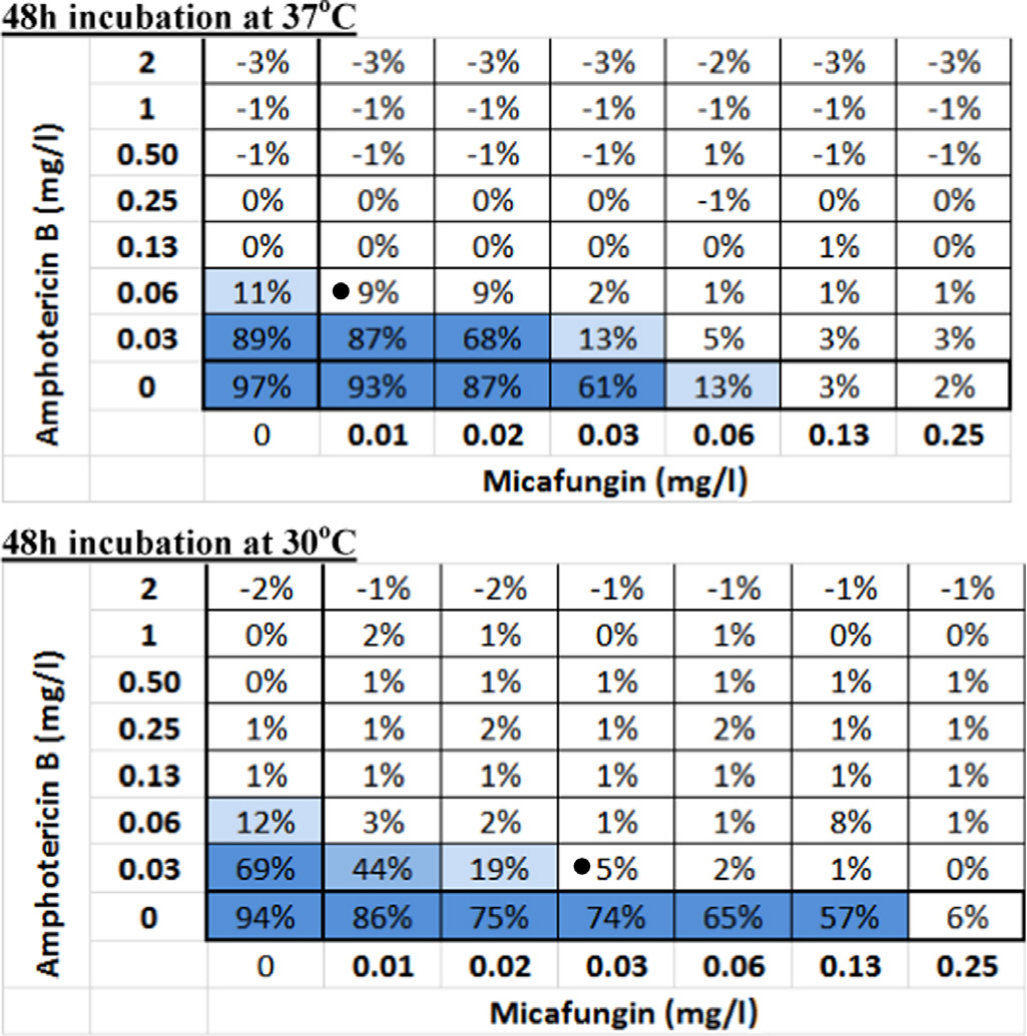
*In vitro* checkerboard of amphotericin B + micafungin after 48 h incubation at 37 °C (top panel) and 30 °C (bottom panel). The Fractional Inhibitory Index using the <10% growth endpoint was 0.56 and 0.375, respectively (black dots).

*In vitro* interaction between amphotericin B and micafungin was determined with a checkerboard broth microdilution as previously described [13]. The combination using 10% growth inhibition endpoint after 48 h at 37 °C and 30 °C was additive and synergistic with a Fractional Inhibitory Index of 0.56 and 0.375, respectively, reducing the MICs of both drugs from 0.125 to 0.06 mg/L for amphotericin B and to 0.01 mg/L for micafungin at 37 °C. A similar decrease was found at 30 °C (Fig. 3). When clear wells were subcultured in order to determine the fungicidal activities the cfu/mL at 0.25 mg/L of amphotericin B were decreased from 2×10^3^ to 0.5×10^3^ cfu/ml when combined with 0.03–0.25 mg/L of micafungin at 30 °C.

## 3 Discussion

*C. pulcherrima* is an environmental, saprophytic yeast but also an opportunistic pathogen. It was isolated from skin lesions and nails [14], [15]. It is member of the *Metschnikowiaceae* family and its morphology and physiology are very close to those of *C. lusitaniae* [14]. This is the second case of neonatal fungemia due to this yeast [16] whereas recently a case of community acquired fungemia caused by *C. pulcherrima* in an injection-drug user was reported [17]. A *C. pulcherrima* blood-stream infection in healthcare setting was related to the use of indwelling catheter for parenteral nutrition [18].

Premature neonates are at particularly increased risk to develop invasive candidiasis with excessive case fatality due to their low birth weight, poor nutrition, enteral malabsorption, insufficient microbial defenses and underdeveloped anatomic barriers. In premature neonates as many as 80% of cases have occurred during the first 42 days of life. Birth weight and postnatal age at the time of infection also predict subsequent mortality. Major risk factors for fungemia include intravascular catheters, parenteral hyperalimentation and broad spectrum antibiotics [1], [4], [6]. In our case prematurity (gestation age of 33 weeks), low birth weight (<2500 g), insufficient immune system and underdeveloped anatomic barriers were the predisposing factors for candidemia. Horizontal transmission via contaminated medical devices, fluids or the hands of health care workers may be the sources [19].

The first case of *C. pulcherrima* fungemia occurred in a neonate by an isolate with fluconazole MIC of 2 mg/L and amphotericin B MIC 0.004 mg/L. It was initially treated with fluconazole (6 mg/kg/d) but because of positive blood cultures, amphotericin B lipid complex (5 mg/kg/d) was initiated. After 6 days of treatment the general condition was improved but only after 15 days of treatment the blood cultures became negative [16]. The second case of *C. pulcherrima* fungemia occurred in an adult by an isolate with caspofungin MIC 0.25 mg/L. It was treated with caspofungin (70 mg/d) and yeast was eradicated at the second day of treatment. Both cases are in line with the present case where amphotericin B failed to eradicate the yeast after 5 days of treatment whereas rapid eradication was observed when micafungin was added. *In vivo* enhancement of liposomal amphotericin B efficacy when combined with micafungin was previously reported for the treatment of azole-refractory *C. guillieremondii* fungemia which failed liposomal amphotericin B plus voriconazole combination therapy [20].

A synergistic effect between amphotericin B and micafungin could explain the rapid eradication when micafungin was added to liposomal amphotericin B therapy. The two-fold reduction of amphotericin B MIC when combined with micafungin could enhance the efficacy of liposomal amphotericin B in order to reach the PKPD target tC_max_/MIC 70 associated with complete response in children. This MIC reduction is particularly important when serum levels are at the lower end of 11–44 mg/L achieved in children with candidemia although neonates may have different pharmacokinetics [21]. In addition, since liposomal amphotericin B is >99% protein bound, serum free concentrations may be lower than the *in vitro* MFC determined in the present study (0.5 mg/L) but sufficient to kill the yeast when combined with micafungin (0.25 mg/L). Persistent *Candida fermentati* fungemia during liposomal amphotericin B therapy was successfully treated previously with the combination of liposomal amphotericin B with caspofungin in a preterm neonate [22]. However, given that the mean duration time of fungal eradication after liposomal amphotericin B therapy of neonatal candidiasis is 9 days [23], the eradication observed in the present study after micafungin was added may be a coincidence with late fungicidal activity of liposomal amphotericin B. Another explanation of rapid eradication could be due to micafungin monotherapy, of which efficacy is shown in clinical trials [24]. Despite the higher MFC, its *in vivo* effect may be enhanced by the strong immunomodulatory effects that echinocandins possess decreasing dysregulated cytokines/chemokines [25]. Finally, since *Candida* species easily form biofilms in catheters, the combination effect may be due to local control of biofilm although in our case no central catheters were present. Although liposomal amphotericin B possesses antibiofilm activity against *C. lusitaniae* at lower concentrations than micafungin (sMIC 0.125 vs >2048 mg/L), micafungin was found to damage more mature biofilms than liposomal amphotericin B at high concentrations achieved locally during infusion [26]. Thus, the combination of liposomal amphotericin B+micafungin may be used for rapid eradication of *C. pulcherrima* in bloodstream infections.

## Conflict of interest

None.
